# The role of hope for health professionals in rehabilitation: A qualitative study on unfavorable prognosis communication

**DOI:** 10.1371/journal.pone.0224394

**Published:** 2019-10-29

**Authors:** Mirjam Amati, Nicola Grignoli, Sara Rubinelli, Julia Amann, Claudia Zanini

**Affiliations:** 1 Department of Health Sciences and Medicine, University of Lucerne and Swiss Paraplegic Research, Lucerne/Nottwil, Switzerland; 2 Sasso Corbaro Medical Humanities Foundation, Bellinzona, Switzerland; 3 Rehabilitation Clinic, Ente Ospedaliero Cantonale, Novaggio, Switzerland; 4 Psychiatry Consultation Liaison Service, Organizzazione Sociopsichiatrica Cantonale, Mendrisio, Switzerland; 5 Swiss Paraplegic Research, Nottwil, Switzerland; National University of Ireland Galway, IRELAND

## Abstract

**Background:**

The communication of prognosis represents an ethical and clinical challenge in medical practice due to the inherent uncertain character of prognostic projections. The literature has stressed that the mode of communicating prognoses has an impact on patients’ hope, which is considered to play a major role in adapting to illness and disability. In light of this, this study aims to explore health professionals’ (HPs) perceptions of the role of hope in rehabilitation and to examine if and how they use strategies to maintain hope when discussing prognostic information with patients.

**Methods:**

Eleven qualitative semi-structured interviews with a purposive sample of HPs were conducted at two rehabilitation clinics in the Canton of Ticino, Switzerland. The interviews were analyzed using thematic analysis.

**Results:**

The HPs perceive hope in rehabilitation as a double-edged sword. Three main strategies were identified to maintain hope while avoiding false hope: 1) giving space for self-evaluation; 2) tailoring the communication of prognostic information; and 3) supporting the patient in dealing with the prognosis. These strategies are particularly suitable when HPs consider that patients might not be ready to accept the prognosis, due to their expectations for recovery.

**Conclusions:**

The strategies identified here support a person-centered approach to the communication of prognosis and are in line with existing protocols for the communication of unfavorable medical information. The findings emphasize the need for strengthening communication and inter-professional collaboration skills of rehabilitation HPs.

## Introduction

The communication of prognosis represents an ethical and clinical challenge in medical practice due to the inherently uncertain nature of prognostic projections [1–3]. Indeed, medical uncertainty is associated with psychological distress for doctors [4] and its communication can be challenging because of the risk that patients perceive doctors as less competent [5]. Moreover, previous studies have shown that when there is little match between the medical prognosis and patients’ expectations for recovery, discussing prognostic information can become even more challenging [6].

In this context, hope is an important factor to be considered. According to positive psychology approaches [7], hope can be defined as an emotion of expectancy that modifies subjective future estimations favorably [8]. Hope has also been described as a “vital resource against despair” and is considered to play a major role in adapting to illness and disability [9]. Besides, maintaining hope is important as hope has been shown to be a key aspect in the recovery process [10–13], influencing the perceived quality of life through expectations [14] and helping people adapt to irreversible changes in their health [15]. Finally, offering hope reflects a patient- or person-centered approach to care [16,17] that is known to positively impact patient outcomes (e.g. satisfaction and self-management) [18] but it can at the same time lead to ethical dilemmas in clinical practice, especially in respecting the right to autonomy [3,19].

Most important the literature has stressed that the mode of communicating prognoses has an impact on patients’ hope [6]. Some authors clearly talk about a “curabo effect”, namely the impact of doctors’ beliefs on patients’ psychosocial outcomes [20]. Nevertheless only little is known about the communication strategies that healthcare professionals use to maintain patients’ hope when disclosing unfavorable prognosis. Much of the literature has focused on how to break bad news in general [21–25] and does not focus on the communication of prognosis specifically.

Existing protocols and guidelines for the communication of unfavorable medical information recognize the need to support hope. For instance, the widely adopted SPIKES protocol in oncology discusses the importance of tailoring the information delivered to the patients by taking into account their hope and expectations [26,27]. Similarly, the ABCDE guidelines [28,29] encourage physicians to “offer realistic hope based on the patient's goals”. However, they do not provide concrete guidance on how to do so and their recommendations are mainly based on a critical review of the existing empirical literature or consensus papers. Furthermore, the majority of the reviewed studies originate from oncology, palliative care and intensive care medicine, whereas other medical fields, such as rehabilitation, have been only marginally studied [30]. Although rehabilitation may deal with patients from across the prognostic spectrum, including end-of-life, in subspecialties like musculoskeletal and neurological rehabilitation, unfavorable prognoses are most often characterized by reduced chances of recovery, with a negative impact on social functioning and subjective health-related quality of life [31–34]. Examples of unfavorable prognoses in this field are aphasia and hemiplegia after ischemic stroke for an elderly patient or decreased functional capacity after a multiple fracture of lower limb caused by a car accident for a young patient. When the prognosis is unfavorable, it is difficult for health professionals to communicate rehabilitation goals in the early phases, knowing that a gap between patients’ hope for recovery and realistic outcome expectations has been found to cause disappointment and influence adherence to treatment or general compliance negatively [10,35].

In light of this, the objective of this study was to explore healthcare professionals’ perceptions of the role of hope in rehabilitation and to examine if and how strategies to maintain hope are used when discussing unfavorable prognostic information with patients.

## Materials and methods

This article presents an explorative qualitative study in the field of rehabilitation.

### Setting

The present study is part of a broader project focused on ethical aspects of the communication of hope in multiple clinical settings (i.e. intensive care, oncology, palliative care, rehabilitation). The findings presented in this article refer to the rehabilitation setting. Data collection was conducted at the two clinics in Southern Switzerland members of the SW!SS REHA network (The Swiss Association of Rehabilitation Clinics), which certifies the most advanced rehabilitation clinics in Switzerland. These clinics were chosen so as to include the entire rehabilitation offer in the region, including neurological and musculoskeletal subspecialties. General data about the patients admitted to the two rehabilitation clinics during the year of the study (i.e. age, gender, route of admission, inpatients/outpatients, objectives set by the Swiss National Association for Quality Development in Hospitals and Clinics (ANQ) [36], Cumulative Illness Rating Scale (CIRS) [37], destination at discharge) were provided by the SW!SS REHA network and are presented in Table 1. These describe the general characteristics of the patients to whom the healthcare professionals participating in this study communicated the prognosis. Data show that the patients were admitted with a mean CIRS severity index of 0.77 and comorbidity index of 3.71, which suggest an average good functional gain and rehabilitation efficiency [38]. Indeed, the vast majority of the patients reached the main ANQ objective set at admission of returning home after rehabilitation. Demographic and clinical data on the population of patients treated in neurological and musculoskeletal rehabilitation clinics in Southern Switzerland during the year of the study are representative of the patients admitted to rehabilitation clinics in Switzerland in the same year [36].

**Table 1:** Data concerning the population of patients admitted to the two rehabilitation clinics divided by subspecialties during the year of data collection.

Data	Neurological	Musculoskeletal	Mixed
Age	67.7	70.2	68.9
Gender	45.6% (female)	63.85% (female)	54.7% (female)
Admission from hospital	74.0%	69.7%	71.2%
Admission from home	25.6%	27.2%	26.6%
Inpatients	622	1233	1855
Outpatients	562	594	1036
ANQ Objective: returning home	91.1%	86.7%	88.9%
CIRS at admission: Comorbidity subscale	4.06	3.36	3.71
CIRS at admission: Severity scale	0.69	0.85	0.77
Destination at discharge: Home	75%	91.7%	86%

### Participants

A purposive heterogeneous sample of doctors and nurses was chosen to ensure that the phenomenon of interest would be explored from different perspectives [39]. Nurses were included because, although the communication of prognostic information is the task of doctors [40,41], in many departments patients and their families identify nurses as key figures for questions and clarification about the prognosis [42–46]. The sample was stratified according to position and experience. For both clinics, we aimed at having at least one doctor with long work experience and one with shorter experience, a head nurse and staff nurse. We imagined that working in different positions, having more or fewer years of work experience and being active in two different rehabilitation fields could play a role at a practical level (involvement in communication; specific skills development) as well as at a conceptual level (individual definition of hope, importance attributed to hope in rehabilitation). The recruitment of participants was based on a snowballing technique: the authors contacted the heads of department who provided the contact details of eligible HPs, who were then contacted by the interviewer (by email or phone).

### Data collection

Data collection was carried out in parallel with preliminary data analysis, and the recruitment of participants was stopped when inductive thematic saturation was reached, namely when additional data did not lead to the development of new themes [47]. Data were collected through face-to-face semi-structured interviews by the first author. Being aware of the potential power imbalance between researcher and participants, we decided to let the latter decide on the interview location and time, so to make them feel at ease in the interview setting. Moreover, the interviewer being an outsider, the participants were in the position of experts, strengthening their feeling of being co-producers, instead of the subject of our research [48].

The interviews took place at the HPs’ rehabilitation clinics during working hours. The interview guide was informed by earlier work [3,6,42,44] and included: general questions about the HPs’ roles in the department and the practice of prognoses communication; questions to explore the role of hope in rehabilitation; questions about barriers and facilitators to maintain hope when communicating an unfavorable prognosis; questions about the participants’ communication skills. The second author was also closely involved in the development of the interview guide because of his knowledge of the settings and of the rehabilitation field. A pilot interview with a doctor was conducted to pre-test the interview guide, which was then modified to improve the flow of the questions and their clarity. Sample questions are presented in Table 2 (See S1 Table for the full Interviews Guide).

**Table 2:** Sample questions for the semi-structured interviews.

Topic	Sample questions
Communication about prognosis	• In your department, who communicates the prognosis? (Do you?) • For you, what is an unfavorable prognosis in rehabilitation? • Do you have any guidelines or rules for communicating prognosis? How do you proceed? • Do you always provide the same information in the same way to patients or do you tailor the communication, and how?
Hope in rehabilitation	• What for you is hope in rehabilitation? • When confronted with an unfavorable prognosis, do you think that hope plays a role for patients? If so, what role?
Barriers and facilitators in maintaining hope when communicating an unfavorable prognosis	• To maintain patients’ hope, what are the difficulties in communicating an unfavorable prognosis? • What do you think can foster hope when you communicate an unfavorable prognosis?
Skills development	• How have you learnt to communicate unfavorable prognoses? (courses at university or continuous education,…) • How do you evaluate your competences in communicating unfavorable prognoses today?

This study was conducted in accordance with the ethical principles for medical research involving human subjects [49] and by paying special attention to the ethical challenges typical of qualitative research [50,51]. Participation in this study was on a voluntary basis and participants had the right to withdraw from the study at any time without explanation. The potential participants were invited to participate, study information was provided and they were given time to decide. All participants provided written informed consent for the semi-structured interviews and their audio recording. This process was especially important considering that the names of potential participants were provided by the heads of department and that some potential participants might have felt under pressure because of the hierarchical relationship. Furthermore, the interviewer was attentive to the potential impact of the research on individual participants and was prepared, for instance, to suggest interrupting the interview if the participant appeared ill-at-ease [52].

We consulted the regional ethics committee, which estimated a minimal risk for our study and concluded that ethical approval was not required.

### Data analysis

The interviews were audiotaped and transcribed verbatim according to the transcription notation system by Braun and Clark [53,54]. Contextual and personal information which could lead to the identification of the participants (e.g., names, names of colleagues, details of workplace such as location, information about professional career) were deleted to ensure confidentiality. The transcripts were analyzed using the principles of thematic analysis.

Thematic analysis is a systematic approach that allows the researcher to identify and report relevant themes or patterns of meaning across the data set [39,54]. Our analysis included both a deductive and an inductive phase. Firstly, the interviews were coded using the topics of the interview guide as a coding scheme. Secondly, the interviews were inductively coded, which means that codes reflecting the content of the data were systematically identified until no new theme emerged. Thirdly, the analysis was refined by collating codes into themes. The themes were subsequently reviewed by comparing all excerpts of the interviews supporting one theme in order to ensure their internal consistency. Finally, the labels of the themes were refined.

The analysis was carried out by two researchers, with the support of the research team. The two researchers do not work in the field of rehabilitation. This had the advantage of approaching the topic with an open perspective and few expectations. The discussions with the health professionals in the research team were very fruitful, as they helped put the information into context.

As suggested by Patton, to enhance the quality and consistency of our analysis, we used analyst triangulation [55]: one researcher took on the role of primary analyst with the second reading all interviews, coding half of them and reviewing the remaining ones. This approach was useful for generating and examining multiple interpretations of the data and for finding convergence [56,57]. The ongoing analysis was discussed within the research team. The two researchers also wrote a research diary to keep track of their discussions and reasoning as well as of their ideas and feelings.

For the coding process, the software MAXQDA© (Release 12.2.0) was used. The analysis was performed on the original interviews by two Italian native-speaker researchers. Relevant quotes were translated into English by a native speaker to support and present the findings in publications. The pseudo-anonymized transcripts of the interviews in the original language (Italian) are available upon request. Detailed information on the consolidated criteria for reporting qualitative research (COREQ) are available (See S2 Table).

## Results

The HPs were five women (nurses) and six men (five doctors and one nurse), ranging in age from 24 to 59 years old. The participants’ characteristics are presented in Table 3. For the sake of confidentiality, we avoided indicating the workplace and the age, as well as presenting the participants grouped by workplace. The interviews lasted an average of 58 minutes.

**Table 3:** Participants’ characteristics.

Profession / Position	Working experience in rehabilitation (years)	Gender
Doctor	20	M
Doctor	13	M
Doctor	12	M
Doctor	5	M
Doctor	2	M
Head nurse	25	F
Head nurse	5	F
Head nurse	2	M
Head Nurse	2	F
Staff nurse	7	F
Staff nurse	<1	F

### HPs’ perceptions of the role of hope in rehabilitation

All HPs stated that hope in rehabilitation is positive because it is associated with patients’ motivation and a higher engagement in the rehabilitation program, which in turn is described as having a positive impact on their functioning. For instance, in the interviews it was mentioned that even the best therapists will be unsuccessful if the patient does not believe that an improvement is possible, as a lack of hope often leads to a lack of commitment to the rehabilitation program or to a treatment. (Table 4, Q1)

**Table 4:** Quotes.

The role of hope in rehabilitation	
1.	“Undoubtedly yes [hope can help] [. . .] we’re also sort of genetically programmed to always find a reason why it’s worth moving forward, but above all we have seen that setting a goal, even a small one, showing the patient that they are not alone and that we can work together to achieve a goal, psychologically this is fundamental and it makes a big difference […]. If you manage to give hope to a patient, they will certainly show considerably more improvement than a patient who is depressed, regardless of their clinical condition.” (Doctor 4 = D4)
2.	“In our area [rehabilitation] I would say the prognosis is quite positive, our patients have a rather high probability of recovering, and resuming their activities at home, their jobs, we are positive in this sense and as a rule we do not have patients with an unfavorable prognosis because basically a rehab patient comes to recover something, to be reintegrated.” (D2)
3.	“The theme of hope, in my opinion, is directly connected with the fact that you rarely have certainty. Medicine by definition is an inexact science with many variables […] and hope is a necessity because it is correct from a psychological and ethical point of view, but it is also a purely practical need due to the fact that even in the worst situations you can give some negative certainties but you are always limited by a high degree of uncertainty, and hope plays in this margin. So basically we say the possibilities go from here as far as here, we are here in the middle, and hope means to make sure that the patient understands that the worst-case scenario might not happen and that there is room for improvement.” (D4)
4.	“[Hope] can be a source of complication if it’s a misplaced hope, that is when hope is not hope but illusion [. . .], when hope becomes something else, that is maybe excessive expectations, as we see here too often with patients and with their families, as if we could make everyone walk, because expectation then leads to disappointment when this expectation is not fulfilled.” (D4)
5.	“It can be positive for a person to leave the clinic using assistive devices for the rest of their life, a wheelchair for instance. […] But it can also be experienced in a negative way by a person who used to run and now needs to use a wheelchair, certainly not ideal, and from his perspective the prognosis would be unfavorable.” (N4)
6.	“In the vast majority of cases patients have the idea that rehabilitation is a treatment that brings them not to the best possible functional state, but back to the same state as before. Whenever you know that you didn’t manage to reach this objective, they [the patients] all have the perception of an unfavorable prognosis. (D3)
7.	“For some patients you need to set limits because they have very high goals, so you have to bring them back to reality. And for some other patients, who might not be depressed in a clinical sense, they need to be encouraged, they could stand up but they are afraid to do so and they don’t have confidence. And then, depending on the type of patient, you have to somehow push a little bit [or] say ‘It is better if you sit because the risk of falling is too high’.” (D3)
8.	“Balancing hope and false hope is very difficult; you need to be intellectually honest with yourself and with your patient.” (D3)
Strategies to give space for self-evaluation	
9.	“Hope is somehow an ideal that a person has in his mind, then there is awareness of reality, and it is our job [as healthcare professionals] to make the two things come closer together. Hope is to say ‘I will go back home and do exactly what I was doing before, bring my goats to the pasture, live alone’. And over time you can start to mention ‘well, maybe the goats need to be sold because it takes two people with assistive devices to lift you and how do you see yourself as a shepherd?’ and the patient slowly [understands].” (D3)
10.	“I think that the positive aspect of being here [in rehabilitation] is that we can confront the patient with his limits. And when he is confronted with his limits, he eventually realizes that his goal is no longer feasible, but it is not me sitting down and out of the blue telling him ‘you won’t be able to climb the stairs anymore’.” (D2)
11.	“Depending on the type of patient, you use different strategies. For instance, with the person who says ‘Don’t worry doctor, when I go home I will cook very well for my family’ you go for a test in the kitchen where he sees that he has difficulty making coffee or that he can make coffee but it takes him 20 minutes.” (D3)
Strategies to tailor the communication of prognostic information	
12.	“I think that to do a good job [communicating] you must know the patient well, this is also the reason why I would not do it right at the beginning of the inpatient stay, but after first understanding the patient, how he is doing, what he knows and what he wants to know, what his background is, what his concerns are, because we don’t know these things on the first day and then with this information we can have a better discussion.” (D2)
13.	“Sometimes this information is gathered by the different healthcare professionals and then we meet once every two weeks and discuss every patient. Because the doctor might not be the point of contact, maybe the patient has concerns that he discusses with the physiotherapist rather than with the neuropsychologist. So the picture of what a person knows, how much he has actually understood and so on can be reconstructed within a few days and from there we start working.” (D3)
14.	“I tend to say that the nurses know a little bit more because they are with the patients twenty-four hours a day seven days a week, at night, during the day, when they feel well and when they are sick, when the relatives are present and when they are alone. The nurses are always there, it is not like the doctor or the physiotherapist who come and go, for this reason nurses have the sensitivity and the knowledge to understand what patients need to know, one might need to know everything, the other only a part of it and to have hope, and the third one needs to know one piece of information now and the next in a week or discover it for herself, because we are all different.” (N3)
15.	“It [the collaborative network between the rehabilitation clinic and the acute hospital] is important. Often, the doctor in the acute hospital has too little experience to know what we do in rehabilitation and how to confront the patient’s questions. By contrast, if the rehabilitation doctor starts seeing the patient in the acute hospital, he can explain how the rehabilitation works; he can eventually dispel the patient’s doubts, and adopt a certain kind of approach in terms of prognosis communication.” (D5)
16.	“If you want to address, for example, such a topic, you need to visit the patient and ask him how everything is going, how he sees the future and then have a discussion about the prognosis, about hope or not, and then to let time pass again. For instance, if the patient indicates that he does not want to speak about the future, I do not know if it is good to communicate the prognosis and say what you think, I think that the patient should slowly come to his own realization.” (D2)
17.	“There is a no time limit ‘after ten days you must [communicate the prognosis]’. We decide at the interdisciplinary team meetings on a case-by-case basis when to meet with the patient and the family, but I would say that there is not one ‘right time’.” (N5)
18.	“[…] procrastinating by giving it [the prognosis] step-by-step is ethical to me […] because there is always something to work on, there are other things that can be done and you slowly get to it [the prognosis].” (D3)
19.	“I try not to do it [giving prognostic information] the first time we meet as I explained, but after at least one week and not at the last visit […]. I try to do it in the middle of the stay in the clinic in case the patient has an emotional shock. So I hear [from physiotherapists, nurses, etc.] what the patient has really understood from what I said to him, and I am always a little surprised. And through this I can gain a lot of insight. But then I go back to the person and I say, ‘Ah I’ve heard that you were affected by my information, I can explain, and we can understand each other.’ And I have to say that this [our discussion] is always well received by patients, and they are happy.” (D2)
20.	“I really appreciate the doctors who say ‘I don’t know’ rather than those who tell you how many months or years you have left, because in the reality every individual is different [. . .]. I think you have to explain to the patient what he has, what is known about the condition and about how it evolves without giving a number, without adding anything else.” (N1)
Strategies to support the patient in dealing with the prognosis	
21.	“In rehabilitation you see these progressions, that the patient improves from day to day and even the patient, when he realizes that he is improving, he gets like a motivational kick, and this is fantastic.” (N4)
22.	“It is a great dispersal of energy thinking about when I will walk [again], if I still cannot control [my trunk]. This should be the goal, we are all working on this, if we get there, we take a next step, if we don’t achieve it, then we have to ask ourselves why we didn’t get there and if we will ever get there. This keeps the patient from deluding himself.” (D3)
23.	“For sure it is important to motivate the patients in the sense of encouraging them when they make progress and not discouraging them when they don’t make progress. [. . .] in my opinion this really helps to increase motivation and hope for improvement.” (N2)
24.	“It is fundamental to show that, regardless of the situation, working is always worthwhile and that we don’t make someone with an unfavorable prognosis feel parked in a bed. When there is an unfavorable prognosis, I don’t lie, I don’t tell you that a miracle will happen, but that it is still worth doing something. The goal in rehabilitation is never only to reach the performance outcome, for instance walking or eating alone. These are all important goals, but the main goal is to try to give the person the best possible quality of life, and this applies to everyone, even to patients with an unfavorable prognosis.” (D4)
25.	“In my opinion a good strategy is to let the patient lead, in the sense that when you don’t know what to say perhaps the best thing to do is just to listen. […] It is more to show that you are there, ready to listen to him if he needs to talk […] and also not to give advice and opinions, to stay really neutral.” (N2)
26.	“In my opinion, you have to explain to the patient what he has [. . .] ((pause)) and above all do not abandon the patient to his own construction of a reality […] if I'm sure of that, what I communicate, also means that I’m responsible for the patient.” (N1)
27.	“We do not express our opinion of the prognosis because it is not our responsibility, but we play an important role in supporting the patient. We support the patient by meeting his needs, and by doing this we improve his condition and also his psychological state.” (N4)
28.	“When you work in a team, you inform the team about the functional status of the patient and the fact that he cannot go home ‘Listen, today I told him that I will send him to an assisted living facility, please, keep an eye on him, he might be crying or he might be inattentive during the exercises because I had to give him bad news.” (D3)

Besides, the participants considered that there is always room for hope in rehabilitation because there is room for improvement. Indeed, the goal of rehabilitation is to achieve and maintain optimal functioning–in the best case scenario, returning to the everyday life one had prior to the injury. (Table 4, Q2) Moreover, one participant stressed that in Switzerland there is a good supportive infrastructure and it is therefore easier to give hope to patients.

The findings also showed that the concept of hope should not be dismissed because medicine is not an exact science and is in continuous development. This means for instance that HPs cannot know with certitude what the rehabilitation outcomes will be and this uncertainty leaves room for patients’ hope. (Table 4, Q3)

However, several HPs thought that hope is problematic when it was a “false hope”, which was defined as an illusion or the unrealistic expectation of a complete recovery process. In this case, hope was described as an obstacle for rehabilitation because, instead of motivating, it becomes a source of disappointment and frustration, and because it does not allow the patient to accept his or her disability. (Table 4, Q4) The interviewees noted also how expectations and hope for recovery could impact the patient reaction to a prognosis, which cannot be defined as favorable or unfavorable based only on a purely medical evaluation of functional recovery. Indeed, the same prognosis can be considered favorable by one patient and unfavorable by another (e.g. a limitation in hand function might not have a big impact on one patient’s life but might change the life of a musician). (Table 4, Q5-Q6) The participants considered that this is very risky for the rehabilitation process, as each time that their expectations are not fulfilled, patients might experience frustration, anger or demotivation.

For this reason, when patients express expectations that are not realistic, HPs try to bring them back within the realm of possibility as described by current medical knowledge, whereas when patients are scared, HPs try to encourage them and support them in going forward. (Table 4, Q7) Hence, HPs have to be very careful in their formulation of the prognosis in order to maintain or foster positive hope and avoid false hope. Finding this balance is very important as this also ensures the maintaining of a relationship of trust between doctor and patient. (Table 4, Q8)

To sum up, hope is considered by the HPs as a double-edged sword: it is simultaneously perceived as having a great potential but also as having risks for the rehabilitation process.

### Strategies to foster or maintain hope (but avoiding false hope)

According to the participants, the prognosis is mostly communicated to patients by doctors and when nurses are confronted with questions regarding prognosis, they refer the patients to doctors. None of the participants received training specific to the communication of prognosis and they were not aware of internal guidelines for this task.

The analysis revealed some of the strategies that the HPs put in place to deal with this situation and maintain or foster hope, and avoid false hope: 1) giving space for self-evaluation; 2) tailoring the communication of prognostic information; and 3) supporting the patient in dealing with the prognosis. These strategies can be used with all patients, but they are particularly relevant when the HPs determine that the patients might not be ready to accept the prognosis, due to their expectations for recovery. The analysis also emphasized how these strategies can be seen as part of a step-by-step approach, in which HPs prepare patients for the communication of the prognosis and support them during and after the communication.

#### Giving space for self-evaluation

Giving space for self-evaluation can be very useful in preparing the patient for the communication of the prognosis. As formulated by a participant, one of the tasks of rehabilitation is to help patients slowly adjust hope to reality, by letting them cognitively digest their new limitations. (Table 4, Q9) In order to do so, the HPs reported giving patients the time to experience their limitations and then involving them in the evaluation of their situation. (Table 4, Q10) This awareness should, on the one hand, facilitate the acceptance of the prognosis and, on the other, limit false hope. For instance, one doctor explained that it is necessary to confront some patients with their limitations by letting them test their capacity in performing daily activities (e.g. cooking, walking up stairs). (Table 4, Q11)

#### Tailoring the communication of prognostic information

The HPs reported that tailoring the communication of the prognosis is helpful in fostering or maintaining patients’ hope, and limiting negative reactions (e.g. frustration, demotivation). In order to do so, the HPs mentioned the importance of collaborative constructing an understanding of the patient as well as of tailoring the timing and the format of the communication.

The HPs stated that tailoring the communication of the prognosis requires information on what patients know, what they understood from previous communications, what they believe, expect and hope. In other words, tailoring requires information on patients’ perspectives on their condition, which HPs can gather during the consultations with them. (Table 4, Q12) To reconstruct the patient’s perspective, the participants indicated that the interdisciplinary team meetings also played an important role, as patients usually discuss their hope and worries with different HPs and only by bringing together this information was it possible to reconstruct the patient’s views precisely. (Table 4, Q13) Moreover, some participants specified that for this task the contribution of nurses is crucial, as they spend more time with patients compared to doctors. By saying this, some nurses emphasized that their contribution and knowledge of patients could be held in greater consideration in order to better understand what patients wish to know. (Table 4, Q14) The importance of having close cooperation with the acute hospitals was also mentioned, as knowing what was communicated previously can help rehabilitation HPs tailor their communication. (Table 4, Q15)

The interviewees also suggested that HPs tailor their communication to patient “readiness”, namely discuss the prognosis when the patient is ready and willing to listen to this information. (Table 4, Q16) According to our participants, the “right moment” could, therefore, vary from patient to patient and is determined on the basis of an assessment of the patient’s condition and situation. (Table 4, Q17) When the prognosis is particularly uncertain, some HPs deemed it acceptable to delay its communication, if this could help the patients preserve hope and motivation for rehabilitation. Uncertainty, indeed, was framed by some HPs as an opportunity to gain time: instead of revealing the prognosis at once, they reported communicating it step-by-step, so as to give the patients time to reach awareness of their situation. (Table 4, Q18) As a general rule, one participant suggested that the best moment to communicate the prognosis is neither at the beginning nor at the end of the hospital stay, but in the middle. In this way, it is possible to support the patient towards acceptance of the prognosis during the time remaining before discharge. (Table 4, Q19)

A few participants explained that they prefer to avoid using percentages (numbers) to describe the patient’s functional status at the end of the rehabilitation program for the reason that some patients might lose hope and be demotivated on hearing low numbers and might invest less in their rehabilitation, therefore compromising their chances of achieving optimal functioning. (Table 4, Q20).

#### Supporting the patient in dealing with the prognosis

Three supportive strategies can be used during and after communication of the prognosis: setting realistic goals, focusing on improvements, monitoring patient reaction for offering support.

To support hope for improvement and commitment to rehabilitation, the participants stressed the importance of working with the patients to set realistic goals that can be reached in the short-term. (Table 4, Q21) Indeed, the HPs reported that, by reaching goals, patients saw the improvements, gained awareness of their own capacities and became motivated to invest in their rehabilitation program. This approach of setting small and realistic goals not only supports motivation but, according to one participant, it avoids wasting energy by focusing on unrealistic or long-term goals. (Table 4, Q22) Because goal-setting necessitates exploring what patients believe, expect and hope, this strategy builds on the understanding of the patient that the HPs have developed.

Furthermore, many participants claimed that helping patients see the glass as half full is also crucial. This included, for instance, focusing on the objectives that have been reached, emphasizing progress instead of the lack of it, with the aim of fostering patients’ motivation and the hope that improvement is possible. (Table 4, Q23) Likewise, some participants stressed the value of always combining communication of the prognosis with a hint of hope, a perspective for the future despite the low likelihood of a complete recovery. The HPs also stated the importance of pointing to what could be improved and of aiming at an enhanced quality of life. (Table 4, Q24)

Finally, the HPs specified that during and after the communication of the prognosis patients need support, emotional support in particular, in order not to lose hope and motivation. The participants explained how they show their engagement with patients, for instance, by acknowledging their experience and taking them seriously or by giving them the chance to ask questions. (Table 4, Q25)

One HP stressed that offering support is a responsibility of HPs and that this is extremely important in order to prevent patients from being deluded. (Table 4, Q26) Whereas doctors are usually the first to discuss the prognosis with patients, support comes from all HPs involved in the rehabilitation. (Table 4, Q27) For this reason, the interviewees considered it important to inform the other team members and cooperate in monitoring the situation and in supporting the patients if need be (e.g. by scheduling an appointment for clarification questions or offering psychological support). (Table 4, Q28)

## Discussion and conclusion

### Discussion

The current study has highlighted doctors’ and nurses’ perception of the role of hope in rehabilitation and identified key communication strategies needed by HPs to foster or maintain hope when communicating unfavorable prognostic information to patients. In the following, we discuss how these findings can contribute to the advancement of the literature on the communication of unfavorable information in the rehabilitation setting.

#### A person-centered approach for fostering hope

First, the analysis stresses that “unfavorable information” in rehabilitation is a matter of subjective quality of life. Although the HPs interviewed in this study agreed that an unfavorable prognosis in rehabilitation has primarily the meaning of functional deficit, they also drew attention to the fact that the interpretation is subjective and largely depends on patients’ needs and goals. This is in line with previous findings stating that bad news is “in the eyes of the beholder” [26] and can be defined as “any information which adversely and seriously affects an individual’s view of his or her future” [58].

Second, as previously outlined by health psychology studies [9,30], our findings indicate that, by enhancing the patients’ engagement in the rehabilitation program, hope might well improve functioning. Hence, maintaining and fostering hope is considered worthwhile by the HPs. Moreover, the findings of our study show how some HPs involved in rehabilitation take advantage of medical uncertainty by delaying the communication of the prognosis or by communicating it step-by-step, if this can help preserve the patient’s hope. Nonetheless, hope needs to be managed to avoid the development of unrealistic expectations and consequent disappointment, which literature has shown can be detrimental for motivation and participation in rehabilitation program [30] or at the moment of discharge [35].

Third, our analysis identified several strategies to foster hope and avoid false hope that HPs working in rehabilitation can use to prepare patients for the communication of the prognosis, to communicate it as well as to help patients deal with the prognostic information. These strategies emphasize a person-centered approach to care [17], with an attempt to involve the patient in evaluating the situation, tailor the different aspects of information provision as well as supporting the patients afterwards.

Moreover, these strategies overlap with the SPIKES protocol in three main points, therefore reaffirming the importance of these steps for the communication of unfavorable information not only in oncology (the field in which they were developed) but also in rehabilitation. First of all, to better tailor the communication, HPs need to understand the patient’s perception of his or her condition (what they think they have, what they have understood from previous communication, etc.). Secondly, before discussing unfavorable prognostic information, HPs should check the patient’s willingness and readiness to receive information. Thirdly, after the communication, it is important to monitor the patient’s reaction to the prognostic information and offer support if needed. Interestingly, in contrast to SPIKES (step 4), our findings do not suggest warning the patient about the bad news (“Unfortunately I’ve got some bad news to tell you”). They suggest, first, to give space for self-evaluation in order to let them experience their situation and adjust hope to reality, and then to communicate the prognosis, if necessary stepwise by adapting the timing to the patient’s readiness to listen. This difference might result from the context in which the SPIKES protocol was developed, namely oncology, a setting in which prognosis can be life-threatening and time an important variable.

#### Inter-professional cooperation and communication

Our findings, however, go a step further by suggesting that these strategies are not only important to facilitate the communication of the prognosis but also to foster and maintain patients’ hope in rehabilitation. Moreover, this study shows that to put into practice some of these strategies, such as tailoring content or monitoring the patient’s reaction and offering support, cooperation among HPs is desirable. This cooperation is very important in rehabilitation, a setting in which different HPs are involved in the treatment of one patient [59–61]. However, similarly to our findings, the literature stresses that nurses wish to be more involved in the communication of prognosis. Empowering nurses in prognosis communication is a key element to improve inter-professional cooperation and the support offered to patients [46]. In some cases, inter-professional and interdisciplinary cooperation can go beyond the confines of the rehabilitation clinic and start in the acute hospital, so as to build a relationship with the patient and better prepare a shared rehabilitation program. To sum up, our findings confirm the importance of understanding the patient’s perspective so as to better tailor the communication and suggest giving patients space for self-evaluation of their functional limitations before communicating the prognosis. This strategy contributes to reconciling the patient’s hope with the reality of the current situation, and therefore setting the stage for fruitful cooperation in the rehabilitation program.

#### Strengths, limitations and future perspectives

This study presents several strengths and limitations. First, although the applicability [62,63] of the findings to other medical settings needs to be corroborated by further studies, the overlaps between our findings and existing guidelines for the communication of unfavorable medical information in other medical fields may indicate a potential applicability of these strategies to other settings. Second, there might have been a (self-) selection bias in the recruitment process: the head doctors at both clinics might have suggested that doctors and nurses with good communication skills or with “unproblematic voices” should participate in the study, or those who agreed to participate might have been the ones more interested in the topic. Third, most of the nurses were female and all the doctors were male. This might have introduced a gender bias. This, however, partially reflects the situation in Switzerland, where most nurses are women and the majority of doctors are male [64]. Finally, this study reports the perspective of HPs active in rehabilitation limited to doctors and nurses; further research is needed to explore and compare the perspectives of others HPs involved in the interdisciplinary team [65]. Additionally, further studies could examine whether the strategies suggested by the participants actually improve rehabilitation outcomes [13].

### Conclusion

The present study highlights hope in rehabilitation as a double-edged sword and identifies strategies used by HPs to foster and maintain patients’ hope in the case of unfavorable prognosis. In this context, it emphasizes the need for a person-centered approach and for inter-professional work to discuss unfavorable prognostic information and to favor hope. Empowering patients in what they can realistically hope for is instrumental in linking perceptions and reality. Ultimately, this study calls for strengthening rehabilitation HPs’ communication and cooperative skills.

## Practice implications

The findings indicate that patients in rehabilitation could benefit from a structured inter-professional cooperation around the communication of prognosis, as their perspective could be better included and the communication tailored accordingly. For this to become possible, doctors and nurses would need specific training in the communication of unfavorable information. Its content could be based on the SPIKES principles and enhanced by the recognition of the role of self-evaluation and hope in rehabilitation. Its format should include standardized tools, simulations and case studies, as suggested by recent work on how to improve inter-professional communication skills [5,38,66]. Barriers to optimal communication regarding prognosis should be assessed before designing interventions. As research has shown, not only inadequate skill and training, but also logistics, clinician discomfort with discussing prognosis, and fear of conflict can impede the implementation of communication protocols [44].

## Supporting information

S1 Table Interview guide. (DOCX)

S2 Table Information on the application of the COREQ (COnsolidated criteria for REporting Qualitative research) checklist. (DOCX)
