# Supplementary material for: The role of hope for health professionals in rehabilitation: A qualitative study on unfavorable prognosis communication
Source: PLoS One. 2019 Oct 29;14(10):e0224394. doi: 10.1371/journal.pone.0224394 (PMC6818780; doi:10.1371/journal.pone.0224394)
Supplement: S1 Table — (DOCX) [file pone.0224394.s001.docx]

S1 Table. **Interviews Guide.**

| Topic | Questions |
| --- | --- |
| Introduction | - Presentation of the interviewer (name, role) and the study. - Introduction to the interview (content, duration, confidentiality). - Request for consent to recording the interview (written consent). |
| Introductory questions | - To begin with, could you tell me something about yourself and describe how your ward / service operates? |
| Communication about prognosis | - In your department, who communicates the prognosis? (Do you?) - For you, what is an unfavorable prognosis in rehabilitation? - How is it decided who should communicate the prognosis? - Do you have any guidelines or rules for communicating the prognosis? How do you proceed? - What prognostic information do you usually communicate to patients? - In your ward are there unfavorable prognoses? Can you give me some examples? - What do you consider an unfavorable prognosis? What are its characteristics? - Article 10 of the ethical code of the FMH^[[1]](#footnote-1)^ states that the doctor is required to give the patient all the information in order that he or she is in a position to decide autonomously for example on possible therapies; at the same time, however, the doctor is required to “assess carefully the way in which he or she intends to inform the patient and how much information the patient is able to bear”. When you have to communicate an unfavorable prognosis:   - How do you assess what and how much information to give?   - Do you have your own recipe, your own way to proceed?   - Do you always provide the same information in the same way to patients or do you tailor the communication, and how?   - Does it happen to you to not communicate the prognosis? For example in what situations?   - Does it happen that the patient asks not to know? If so, what do you do? |
| Hope in rehabilitation | - Have you ever thought about hope in connection with the prognosis? - What for you is hope in rehabilitation? - When confronted with an unfavorable prognosis, do you think that hope plays a role for patients? If so, what role? - Do you think that hope can also be negative? - Can the way an unfavorable prognosis is communicated influence the patient’s hope? If so, do you have some strategy for keeping alive the patient’s hope? What? - In your opinion, is there a right time to communicate an unfavorable prognosis? - In you opinion, can medical uncertainty concerning the prognosis influence hope? How? For what reason? - Do you have some strategies for communicating uncertainty while maintaining hope? |
| Barriers and facilitators in maintaining hope when communicating an unfavorable prognosis | - To maintain patients’ hope, what are the difficulties in communicating an unfavorable prognosis? - What do you think can foster hope when you communicate an unfavorable prognosis? |
| Skills development | - How have you learnt to communicate unfavorable prognoses? (courses at university or continuous education,…)   - If you have taken courses, was this during basic or continuous training?   - If while working, did you have a mentor or did you proceed by experimenting? - How do you rate your competences in communicating unfavorable prognoses today? - How do you live the communication of an unfavorable prognosis? |
| Conclusion | - In conclusion, is there anything else that you think is important but that we haven’t discussed and that you would like to add? - Do you have any questions? - Thanks. - Switch off the recorder. - Explain the next steps. |

1. FMH (2018) Art. 10. Code of Ethics of the FMH. Bern. [↑](#footnote-ref-1)
