# Supplementary material for: The role of hope for health professionals in rehabilitation: A qualitative study on unfavorable prognosis communication
Source: PLoS One. 2019 Oct 29;14(10):e0224394. doi: 10.1371/journal.pone.0224394 (PMC6818780; doi:10.1371/journal.pone.0224394)
Supplement: S2 Table — (DOCX) [file pone.0224394.s002.docx]

S2 Table. **Information on the application of the COREQ checklist.**

| **Domain 1: Research team and reflexivity** |
| --- |
| **Personal Characteristics**  1. Interviewer: Which author conducted the interviews?  🡪 *First author (MA). See section 2.3 in the manuscript.*  2. Credentials: What were the researcher’s credentials? E.g. PhD, MD  🡪 *Master of Arts (for the interviewer: MA), PhD (for all the co-authors: NG, SR, JA, CZ)*  3. Occupation: What was their occupation at the time of the study?  🡪 *MA: intern at a research institution; NG: researcher and health psychologist; SR, JA, CZ: researchers*  4. Gender: Was the researcher male or female?  🡪 *The interviewer was female. The team was composed of female and male researchers.*  5. Experience and training: What experience or training did the researcher have?  🡪 *MA was trained during the Master’s program and her internship. NG was trained during his Master’s and doctoral degree programs. SR, JA and CZ have extensive experience in the conduction of qualitative research.*  **Relationship with participants**  6. Relationship established: Was a relationship established prior to study commencement?  🡪 *A relationship was established by email or phone for presenting the study and discussing participation in it.*  7. Participant knowledge of the interviewer: What did the participants know about the researcher? e.g. personal goals, reasons for doing the research  🡪 *The interviewer introduced herself at the beginning of the interview.*  8. Interviewer characteristics: What characteristics were reported about the interviewer? E.g. bias, assumptions, reasons and interests in the research topic  🡪 *The interviewer was expecting that the work experience (in terms of position and role) would make a difference in her perspective. This is reflected in the sampling strategy.* *See “Participants” section in the manuscript.* |
| **Domain 2: study design** |
| **Theoretical framework**  9. Methodological orientation and theory: What methodological orientation was stated to underpin the study? e.g. grounded theory, discourse analysis, ethnography, phenomenology, content analysis  🡪 *No specific theoretical framework underpinned the analysis.*  **Participant selection**  10. Sampling: How were participants selected? e.g. purposive, convenience, consecutive, snowball  🡪 *Purposive sampling. See “Participants” section in the manuscript.*  11. Method of approach: How were participants approached? e.g. face-to-face, telephone, mail, email  🡪 *By email or phone. See “Participants” section in the manuscript.*  12. Sample size: How many participants were in the study?  🡪 *Eleven participants. See “Results” section in the manuscript.*  13. Non-participation: How many people refused to participate or dropped out? Reasons?  🡪 *None.*  **Setting**  14. Setting of data collection: Where was the data collected? e.g. home, clinic, workplace  🡪 *Hospitals. See “Setting” and “Data collection” sections in the manuscript.*  15. Presence of non-participants: Was anyone else present besides the participants and researchers?  🡪 *No.*  16. Description of sample: What are the important characteristics of the sample? e.g. demographic data, date  🡪 *A description of the sample is provided in “Results” section (introduction to the results) and in Table 3. More details were not given to ensure confidentiality,*  **Data collection**  17. Interview guide: Were questions, prompts, guides provided by the authors? Was it pilot tested?  🡪 *Yes. See sample question in Table 2 in the manuscript. The interview guide was pilot tested (see section “Data collection” in the manuscript).*  18. Repeat interviews: Were repeat interviews carried out? If yes, how many?  🡪 *No.*  19. Audio/visual recording: Did the research use audio or visual recording to collect the data?  🡪 *Audio recording and verbatim transcription. See section “Data analysis” in the manuscript.*  20. Field notes: Were field notes made during and/or after the interview?  🡪 *The interviewer took notes after each interview concerning her first impression, her thoughts, , her feelings as well as potential links to other interviews, ideas for analysis, etc.*  21. Duration: What was the duration of the interviews?  🡪 *On average 58 minutes. See section “Results" in the manuscript.*  22. Data saturation: Was data saturation discussed?  🡪 *Yes. See section “Data collection” in the manuscript.*  23. Transcripts returned: Were transcripts returned to participants for comment and/or correction?  🡪 *No.* |
| **Domain 3: analysis and findings** |
| **Data analysis**  24. Number of data coders: How many data coders coded the data?  🡪 *Two coders (first and last authors), and additional discussion with the research team. See “Data analysis” section in the manuscript.*  25. Description of the coding tree: Did authors provide a description of the coding tree?  🡪 *No.*  26. Derivation of themes: Were themes identified in advance or derived from the data?  🡪 *Both a deductive and an inductive approach. See “Data analysis” section in the manuscript.*  27. Software: What software, if applicable, was used to manage the data?  🡪 *MAXQDA. See “Data analysis” section in the manuscript.*  28. Participant checking: Did participants provide feedback on the findings?  🡪 *No.*  **Reporting**  29. Quotations presented: Were participant quotations presented to illustrate the themes / findings? Was each quotation identified? e.g. participant number  🡪 *Yes. See “Results” section in the manuscript.*  30. Data and findings consistent: Was there consistency between the data presented and the findings?  🡪 *Yes. See “Results” section in the manuscript.*  31. Clarity of major themes: Were major themes clearly presented in the findings?  🡪 *Yes. See “Results” section in the manuscript.*  32. Clarity of minor themes: Is there a description of diverse cases or discussion of minor themes?  🡪 *Yes. See “Results” section in the manuscript.* |
